# HOXA11 hypermethylation is associated with progression of non-small cell lung cancer

**DOI:** 10.18632/oncotarget.1464

**Published:** 2013-10-28

**Authors:** Jung-Ah Hwang, Bo Bin Lee, Yujin Kim, Seong-Eun Park, Kyun Heo, Seung-Hyun Hong, Young-Ho Kim, Joungho Han, Young Mog Shim, Yeon-Su Lee, Duk-Hwan Kim

**Affiliations:** ^1^ Cancer Genomics Branch, Research Institute, National Cancer Center, Goyang-si, Korea; ^2^ Department of Molecular Cell Biology, Samsung Biomedical Research Institute, Sungkyunkwan University School of Medicine, Suwon, Korea; ^3^ Department of Internal Medicine, Samsung Medical Center, Sungkyunkwan University School of Medicine, Seoul, Korea; ^4^ Department of Pathology, Samsung Medical Center, Sungkyunkwan University School of Medicine, Seoul, Korea; ^5^ Department of Thoracic and Cardiovascular Surgery, Samsung Medical Center, Sungkyunkwan University School of Medicine, Seoul, Korea

**Keywords:** HOXA11, Hypermethylation, Non-small cell lung cancer, Progression, Migration

## Abstract

This study was aimed at understanding the functional significance of *HOXA11* hypermethylation in non-small cell lung cancer (NSCLC). *HOXA11* hypermethylation was characterized in six lung cancer cell lines, and its clinical significance was analyzed using formalin-fixed paraffin-embedded tissues from 317 NSCLC patients, and Ki-67 expression was analyzed using immunohistochemistry. The promoter region of *HOXA11* was highly methylated in six lung cancer cell lines, but not in normal bronchial epithelial cells. The loss of expression was restored by treatment of the cells with a demethylating agent, 5-aza-2'-deoxycytidine (5-Aza-dC). Transient transfection of *HOXA11* into H23 lung cancer cells resulted in the inhibition of cell migration and proliferation. *HOXA11* hypermethylation was found in 218 (69%) of 317 primary NSCLCs. *HOXA11* hypermethylation was found at a higher prevalence in squamous cell carcinoma than in adenocarcinoma (74% vs. 63%, respectively). *HOXA11* hypermethylation was associated with Ki-67 proliferation index (*P* = 0.03) and pT stage (*P* = 0.002), but not with patient survival. Patients with pT2 and pT3 stages were 1.85 times (95% confidence interval [CI] = 1.04-3.29; *P* = 0.04) and 5.47 times (95% CI = 1.18-25.50; *P* = 0.01), respectively, more likely to show *HOXA11* hypermethylation than those with pT1 stage, after adjusting for age, sex, and histology. In conclusion, the present study suggests that *HOXA11* hypermethylation may contribute to the progression of NSCLC by promoting cell proliferation or migration.

## INTRODUCTION

Lung cancer is currently the leading cause of cancer-related deaths worldwide despite significant strides in its early detection and treatment over the past few decades; the overall 5-year survival rate remains low at 10% - 15% [1]. Micrometastasis, which has occurred in over two-thirds of patients at diagnosis, along with the high rate of recurrence after resection, is mainly responsible for the poor prognosis of lung cancer patients. Even those patients with stage 1 lung cancer detected prior to the spread of the cancer to other nearby lymph nodes and sites still suffer from poor prognosis [2]. Hence, it is vital to develop biomarkers to be used for the identification of patients with a relatively high risk of poor prognosis and for the early detection and targeted therapy. Over the past few years, several epigenetic biomarkers have been developed with this goal in mind.

*HOX* genes encode transcription factors that play essential roles in embryonic development and differentiation of adult cells. *HOX* genes are also known to play an essential role in lung development and are expressed in the normal human adult lung [3]. *HOX* genes in mammals are arranged into clusters (A, B, C, and D) on four different chromosomes [4]. The *HOXA* cluster, located within a 155-kb-long genomic region on chromosome 7p15-7p14.2 consists of 12 genes (11 *HOX* genes and EVX1) [5]. Highly dense CpG islands are prevalent in most of the *HOXA* promoters and the hypermethylation of these islands play pivotal roles in the control of *HOXA* gene expression. Among *HOX*A genes, *HOXA11* hypermethylation has recently been reported in lung cancer [6-8], ovarian cancer [9, 10], glioblastoma multiforme [11], follicular lymphoma [12], endometrial adenocarcinoma [13] and cervical cancer [14]. Nonetheless, the clinicopathological significance of its methylation remains to be uncovered for lung cancer, and *HOXA11* hypermethylation is currently a target of active research.

To gain better insight into the role of *HOXA11* gene in NSCLCs, we characterized the *HOXA11* hypermethylation *in vitro* and further investigated the association between clinicopathological parameters and *HOXA11* hypermethylation in paraffin-embedded tissues from 317 primary non-small cell lung cancers (NSCLCs).

## RESULTS

### Methylation analysis of *HOXA11* promoter *in vitro*

**Figure 1:**
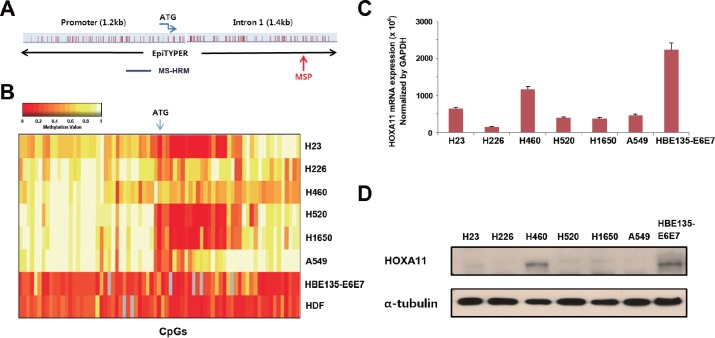
Analysis of the methylation and expression of *HOXA11 in vitro* (A) Gene map shows location of CpGs that were studied by different methods in this study. (B) The methylation status of 90 CpGs at the promoter region of *HOXA11* was analyzed using the EpiTYPER™ assay in six lung cancer cell lines (H23, H226, H460, H520, H1650, and A549), a bronchial epithelial cell line (HBE135-E6E7), and in a normal human dermal fibroblast (HDF). Two-way cluster analysis shows the methylation status of *HOXA11* in eight cell lines. Levels of methylation are depicted in color change on a continuous scale from red (0% methylated) to light yellow (100% methylated). X-axis and Y-axis indicate CpG sites and cell lines, respectively. (C & D) The mRNA levels of HOXA11 were analyzed by real-time PCR (C), and protein levels were determined using western blotting (D). Error bars indicate one standard deviation.

The locations of CpGs that were analyzed using EpiTYPER™, MS-HRM, and MSP are indicated in Figure 1A. The *HOXA11* promoter sequence was obtained from Transcription Element Search System (http://www.cbil.upenn.edu/cgi-bin/tess/tess), and methylation statuses of 90 CpGs at the promoter region of *HOXA11* were first analyzed quantitatively using the EpiTYPER™; some of the CpGs were partially methylated in H23, H520, and H1650 cells (Fig. 1B). *HOXA11* expression, analyzed using quantitative real-time PCR (Fig. 1C) and western blotting (Fig. 1D), correlated well with these methylation statuses. The mRNA and protein levels of six lung cancer cell lines were downregulated compared to HBE135-E6E7 bronchial epithelial cells, except weak expression in H460. This result suggests that *HOXA11* hypermethylation may be responsible for silencing of *HOXA11*.

### 5-Aza-dC induced demethylation and re-expression of silenced *HOXA11*

**Figure 2:**
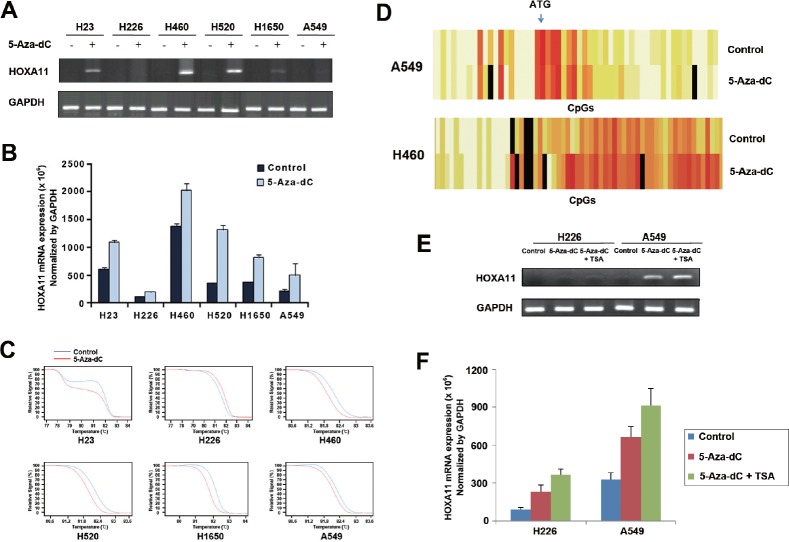
Effects of 5-Aza-dC and/or TSA on demethylation and re-expression of silenced *HOXA11* (A & B) Re-expression of silenced HOXA11 was examined by RT-PCR (A) and real-time PCR (B) in six lung cancer cell lines after treatment of the cells with 5-Aza-dC for 72 h. Compared to other cell lines, re-expression of HOXA11 is minimal in H226 and A549 cells. Plus and minus signs indicate the presence or absence of 5-Aza-dC treatment, respectively. (C) Demethylation of silenced HOXA11 was analyzed using MS-HRM assay after treatment of 5-Aza-dC for 72 h in 6 lung cancer cell lines. Red and blue lines indicate the statuses with and without treatment of 5-Aza-dC, respectively. (D) Heatmaps show typical patterns of demethylation: the degree of demethylation is high in H460 cells with less densely methylated *HOXA11* and minimal in A549 cells with densely methylated *HOXA11*. (E & F) H226 and A549 cells that showed minimal re-expression of HOXA11 were treated with 10 M 5-Aza-dC in combination with 0.5 M TSA for 72 h. TSA induced additional re-expression of HOXA11 by RT-PCR (E) and real-time PCR (F). Error bars indicate one standard deviation.

Dependence of the downregulation of *HOXA11* in lung cancer cells on hypermethylation of the gene was validated by analyzing the re-expression and demethylation of silenced genes using RT-PCR (Fig. 2A), quantitative real-time PCR (Fig. 2B), MS-HRM assay (Fig. 2C), and EpiTYPER™ (Fig. 2D), after treatment of lung cancer cells with 10 μM 5-Aza-dC for 72 h. Re-expression of *HOXA11* (Figs. 2A and 2B) in response to 5-Aza-dC was minimal in H226 and A549 cells, but other cell lines showed a substantial increase at the mRNA levels of *HOXA11*. The degree of demethylation after treatment of 5-Aza-dC was also minimal in H226 and A549 cells (Fig. 2C). Figure 2D shows a typical pattern of demethylation: demethylation occurred much more in H460 cells with less densely methylated CpGs than in A549 cells with densely methylated CpGs, suggesting that the degree of demethylation in response to 5-Aza-dC may be inversely related to the density of initial hypermethylation. Because histone deacetylase inhibitor is known to cooperate for re-expression of hypermethylated genes, the H226 and A549 cells showing minimal re-expression of silenced *HOXA11* in response to 5-Aza-dC were further co-incubated with 5-Aza-dC for another 24 h in the presence of 0.5 μM TSA following 48 h of initial 5-Aza-dC treatment. TSA along with 5-Aza-dC in H226 and A549 cells induced reactivation of the silenced *HOXA11* at the level of mRNA by RT-PCR (Fig. 2E) and real-time PCR (Fig. 2F).

### *HOXA11* inhibited cell migration and proliferation in lung cancer cells

**Figure 3:**
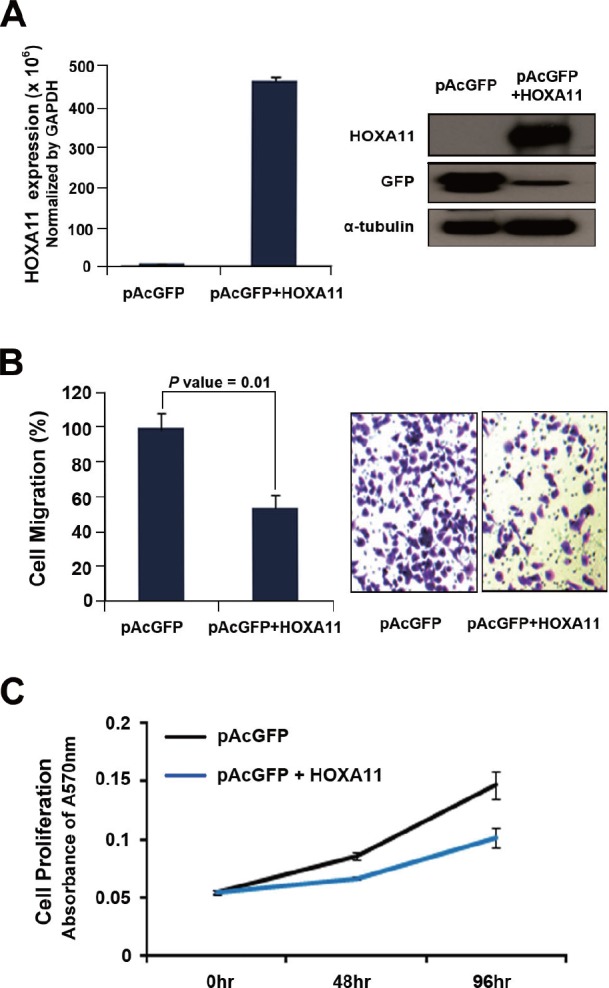
The effect of *HOXA11* on cell migration and proliferation (A) To analyze the effect of HOXA11 on cell migration and proliferation, H23 lung cancer cells were transfected with pAcGFP-HOXA11 fusion construct, or empty vector. After transfection, the expression of HOXA11 was confirmed by western blot using primary antibody directed to GFP (sc-9996; Santa Cruz Biotechnology, CA, USA). (B) H23 cells transfected with pAcGFP-HOXA11 were re-seeded in 6.5 mm Transwell® with 8 μm pore size (Corning, USA). The cells were induced to migrate into 1% of FBS containing media in 24 well plate. After 48 hr each transwell insert was stained by 1% crystal violet and destained with 10% acetic acid. The absorbance was measured at 564 nm using VERSAmax microplate reader (Molecular devices). (C) H23 cells transfected with pAcGFP-HOXA11 were seeded in a 96 well plate, and cell proliferation activity was measured every 24hrs with MTT assay. Y axis indicates absorbance measured at 570 nm using VERSAmax microplate reader (Molecular devices), and the data are presented as mean ± standard error (SE) of eight experiments.

To investigate the function of *HOXA11* in tumorigenesis, cell migration and cell proliferation was analyzed in H23 cells induced by transient transfection of GFP-tagged *HOXA11*. Anti-GFP antibody was used to detect the expression of *HOXA11* (Fig. 3A) by western blot analysis. Cell migration was significantly reduced in H23 cells transfected with pAcGFP-*HOXA11* construct (P = 0.01; Fig. 3B). Cell proliferation was also analyzed in the H23 cells after transfection of the GFP-fusion constructs. Cell proliferation was inhibited in H23 cells induced by pAcGFP-*HOXA11* (Fig. 3C): pAcGFP-*HOXA11* suppressed cell proliferation by 30% at 72 h after seeding the cells for MTT assay. Based on these observations, it is likely that *HOXA11* may function as a tumor suppressor by inhibiting cell migration and cell proliferation in tumorigenesis of the lung.

### Association of *HOXA11* hypermethylation with clinicopathological variables in primary NSCLCs

**Figure 4:**
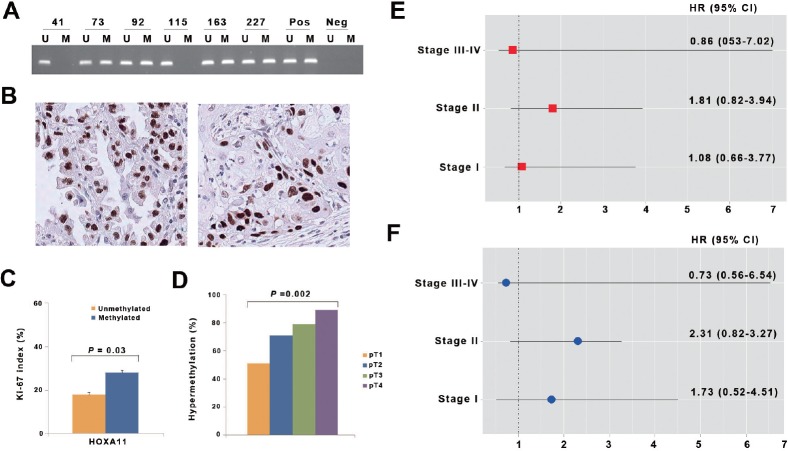
Analysis of *HOXA11* hypermethylation in primary non-small cell lung cancer (A) Promoter hypermethylation of *HOXA11* was analyzed in paraffin-embedded tissues from 317 NSCLC patients using methylation-specific PCR. Patient identification numbers are indicated. “Pos” represents the positive controls for the methylated (M) and unmethylated (U) allele. Negative control samples without DNA were included for each PCR. (B) Expression of Ki-67 was analyzed using immunohistochemistry. Figures show representative examples of positive expression of Ki-67 in adenocarcinoma (left) and squamous cell carcinoma (right). (C) Ki-67 proliferation index was compared according to methylation statuses of *HOXA11*. Error bars indicate standard error. (D) The prevalence of *HOXA11* hypermethylation was calculated across pT lesion. *HOXA11* hypermethylation showed a significant association with pT lesion (*P* = 0.002). (E & F) The adjusted hazards ratios (HRs) and 95% confidence intervals (CIs) for death (E) and recurrence (F) are illustrated across pathologic stage, after controlling for potential confounding factors.

In order to understand the clinicopathological significance of *HOXA11* hypermethylation, we analyzed the methylation status of *HOXA11* using methylation-specific PCR (Fig. 4A) and the Ki-67 proliferation index using immunohistochemistry (Fig. 4B) in the 317 NSCLCs with a median follow-up period of 5.2 years. The relationship between clinicopathological characteristics and *HOXA11* hypermethylation is described in Table 1. Hypermethylation of *HOXA11* was found in 218 (69%) of 317 patients studied. *HOXA11* hypermethylation was not associated with patient age, sex, exposure to tobacco, differentiation, pathologic stage, histology, pathologic stage, and tumor recurrence. However, the Ki-67 proliferation index was significantly different according to the methylation status of *HOXA11* (Fig. 4C). The mean of the Ki-67 proliferation index was 28% and 19% in tumors with and without hypermethylation of *HOXA11*, respectively, and the difference was statistically significant (P = 0.03). In addition, *HOXA11* hypermethylation was significantly associated with pT stage (Fig. 4D). *HOXA11* hypermethylation was noted to occur in 32 (51%) of the 63 patients with pT1 stage, 154 (71%) of the 216 with pT2 stage, 15 (79%) of the 19 with pT3 stage, and 17 (89%) of the 19 diagnosed with pT4 stage (P = 0.002).

**Table 1:** Clinicopathological characteristics (N=317)

Variables	*HOXA11* hypermethylation		*P*-value
	No (N=99)	Yes (N=218)	
Age*	60 ± 10	60 ± 10	0.73
Pack-years*	32 ± 40	32 ± 27	0.23
Sex			
Women	29	53	
Men	70	165	0.35
Smoking status			
Never	29	66	
Former	12	36	
current	58	116	0.54
Histology			
Adenoca	46	78	
Squamous	44	121	
Others	9	19	0.17
Differentiation^†^			
Well	21	33	
Moderately	40	108	
Poorly	13	19	
Undifferentiated	2	5	0.24^‡^
Stage			
I	63	124	
II	25	53	
III	11	37	
IV	0	4	0.33^‡^
Recurrence			
No	50	124	
Yes	49	94	0.29

Abbreviations: Adenoca, adenocarcinoma; Squamous, squamous cell carcinoma;

* Values indicate mean ± standard deviation

† Differentiation data are missing for 76 patients

‡ Based on Fisher's exact test

**Table 2:** Logistic regression analysis* of HOXA11 hypermethylation

	OR	95% CI	P-value
Histology			
Adenoca	1.00		
Squamous	1.71	1.01-2.89	0.03
pT			
pT1	1.00		
pT2	1.85	1.04-3.29	0.04
pT3	5.47	1.18-25.50	0.01
pT4	5.85	1.21-27.28	0.01

Abbreviations: adenoca, adenocarcinoma; squamous, squamous cell carcinoma; OR, odds ratio; CI, confidence interval

* Adjusted for age and sex

Logistic regression analysis of the data was performed to control for the potential confounding effects of variables such as age, sex, and histology. *HOXA11* hypermethylation occurred at a 1.85 times (95% confidence interval [CI] = 1.04-3.29; P = 0.04) higher prevalence in pT2 stage than in pT1 stage, after adjusting for age and histology. Patients with pT3 stage were 5.47 times (95% CI = 1.18-25.50; P = 0.01) more likely to show *HOXA11* hypermethylation than those with pT1 stage. Finally, the relationships between hypermethylation of *HOXA11* and patient's overall survival and recurrence-free survival were analyzed across pathologic stage. However, no association was found between *HOXA11* hypermethylation and patient's survival (Figs. 4E and 4F).

## DISCUSSION

*HOXA11* encodes a DNA-binding transcription factor, which may regulate gene expression, morphogenesis, and differentiation. *HOXA11* hypermethylation has been reported in lung cancer [6-8], but its functional relationship with malignant phenotype in lung cancer has remained elusive. Several examples exist in which homeobox genes impinge on the cell cycle, but it is not clear how *HOXA11* hypermethylation contributes to carcinogenesis of lung. In this study, *HOXA11* hypermethylation was found at a higher prevalence in squamous cell carcinoma than adenocarcinoma and occurred at approximately 2 to 5 times higher prevalence in pT2–pT4 stage than pT1 stage. *HOXA11* hypermethylation was also associated with Ki-67 proliferation index in 317 paraffin-embedded tissues, and transfection of pAcGFP-*HOXA11* into H23 cells inhibited the migration and proliferation of the cells. These observations suggest that *HOXA11* may function as a tumor suppressor by inhibiting cell proliferation and migration in lung cancer.

It is not clear what is responsible for *HOXA11* hypermethylation in NSCLC. Increased activity of DNA methyltransferase is one of the possible factors responsible for increased susceptibility to aberrant methylation of CpG island at the promoter region of tumor suppressor genes [15, 16]. Smoking is known to increase the activity of DNA methyltransferase, and thereby induces *de novo* methylation. Increased levels of DNMT1 were observed in the lung of A/J mice exposed to the tobacco specific carcinogen 4-methylnitrosamino-1-(3-pyridyl)-1-butanone (NNK) [17]. In addition, several groups have reported positive relationship between exposure to tobacco smoke and CpG island hypermethylation in NSCLCs. These observations support that the hypermethylation of CpG island at a gene may be regulated by smoking [18-20]. Therefore, we analyzed the association between exposure to tobacco smoke and *HOXA11* hypermethylation in patients. In addition, to rule out the possibility of age-dependent hypermethylation of *HOXA11* in NSCLC patients, we analyzed the association between *HOXA11* hypermethylation and patient age. However, no association was found between exposure to tobacco smoke or patient age and *HOXA11* hypermethylation, irrespective of histology (data not shown). Further study is needed to understand the underlying mechanism of *HOXA11* hypermethylation in NSCLC.

DNA methylation and histone deacetylation are known to work synergistically to establish and maintain a repressive chromatin state and to silence gene transcription. In this study, the demethylation and reactivation of *HOXA11* in response to a low dose of 5-Aza-dC was detected for the majority of the cell lines but minimally for H226 and A549 cells (Figs. 2C and 2D). As it is known that histone deacetylase inhibition by TSA facilitates transcriptional reactivation induced by 5-Aza-dC, TSA treatment resulted in slight re-expression of the *HOXA11* gene to a level detectable by RT-PCR in A549 cells and by real-time PCR in H226 cells (Figs. 2E and 2F). It was not clear what was responsible for low response of *HOXA11* to 5-Aza-dC and TSA in H226 cells. However, there may be several possibilities. First, the patterns of alterations of gene expression induced by different classes of HDACIs are known to be different in various transformed cells. Accordingly, TSA might not be effective in leading to a more relaxed chromatin conformation in H226 cells, and other classes of HDACIs may be required for the re-expression of *HOXA11* in H226 cells. Second, the failure in reversal of repressive transcriptional chromatin modifications following 5-Aza-dC treatment could be due to the short time course of the experiments and low dose of 5-Aza-dC. Finally, *HOXA11* in H226 cells may be inactivated by another mechanism rather than by DNA methylation.

Finally, we analyzed the effect of *HOXA11* hypermethylation on patient prognosis. The effect of *HOXA11* hypermethylation on overall survival and recurrence-free survival was analyzed in 317 patients. There is no report about the prognostic significance of *HOXA11* hypermethylation in lung cancer, and no association was found between *HOXA11* hypermethylation and recurrence-free survival or overall survival in this study. However, some have reported prognostic significance of *HOXA11* hypermethylation in other types of cancers: *HOXA11* hypermethylation is more frequent in recurrent endometrial adenocarcinoma than in non-recurrent endometrial adenocarcinoma and is associated with poor prognosis in early stage endometrial adenocarcinoma. *HOXA11* hypermethylation is also known to be strongly associated with the residual tumor after cytoreductive surgery in ovarian cancer, and with poor outcome [9]. In addition, *HOXA11* hypermethylation was detected in normal endometrium from premenopausal ovarian cancer patients, suggesting that *HOXA11* hypermethylation can be a predictive marker for ovarian cancer [10]. Based on these observations, it is likely that the effect of *HOXA11* hypermethylation on patient survival is tissue-specific.

This study had several limiting actors. First, silencing by DNA methylation usually involves methyl-binding domain proteins binding to the DNA and recruiting histone deacetylases (HDACs). Therefore, the DNA binding and HDACs recruiting to the plasmid and the nucleosome assembling on a transfected plasmid needed to be studied for understanding the mechanism underlying transcriptional silencing by *HOXA11* hypermethylation. Second, The effect of *HOXA11* on cell migration and proliferation was performed in only H23 lung cancer cells. Considering large heterogeneity between established cell lines, it needs further experiments in other cell lines. In addition, *HOXA11* was associated with tumor cell migration and proliferation. However, their downstream targets are still unknown; delineation of the target genes regulated by *HOXA11* is required. Finally, 500-bp upstream from the ATG translation start codon (Fig. 1B) may be associated with transcriptional downregulation by *HOXA11* hypermethylation. Further study is warranted to find critical CpGs responsible for transcription. In conclusion, the present study suggests that *HOXA11* hypermethylation may be involved in the progression of NSCLC through increased cell proliferation and migration.

## MATERIALS AND METHODS

### Cell culture

Six human lung cancer cell lines (H23, H226, H460, H520, H1650, A549), a human bronchial epithelial cell line (HBE135-E6E7), and a human dermal fibroblast (HDF) were obtained from the American Type Culture Collection (Manassas, VA). The cells were grown in a designated growth media supplemented with 10% heat-inactivated fetal bovine serum (Hyclone, Logan, UT) and 1% antibiotic-antimycotic (Gibco, New York, NY), and were maintained at 37°C in an atmosphere of 5% CO2.

### Tissue samples

Formalin-fixed paraffin-embedded tumor tissues were collected from 317 NSCLC patients who underwent curative surgical resection between August 1994 and November 2011 at the Department of Thoracic and Cardiovascular Surgery, Samsung Medical Center, Seoul, Korea. Post-operative follow-up for survival or recurrence was conducted as previously described [2]. The pathological stage of each specimen was determined according to the guidelines of the AJCC TNM staging system [21]. Written informed consent for use of tissues resected, as approved by the Institutional Review Board at the Center, was obtained from each patient prior to surgery.

### Genomic DNA extraction and sodium bisulfite modification

Genomic DNA from cultured cells and paraffin-embedded tissues was extracted using the QIAamp DNA Mini Kit (Qiagen, Hilden, Germany) and DNeasy Tissue kit (Qiagen, Valencia, CA), respectively, according to the manufacturer's protocols. One microgram of genomic DNA from each sample was modified with sodium bisulfite using the EZ DNA Methylation-Gold Kit (ZYMO Research, Irvine, CA) according to the manufacturer's instructions. Areas containing at least 75% or more of neoplastic cells in the paraffin-embedded tissues were used in this study.

### Quantitative Analysis of methylation using EpiTYPER

Methylation statuses of 90 CpGs at the promoter region of *HOXA11* were evaluated in six lung cancer cell lines, HBE135-E6E7 cells, and HDF cells by using the EpiTYPER™ (Sequenom, San Diego, CA); methylation primers (Supplementary Table S1) for bisulfite-converted DNA were designed using EpiDesigner software (www.epidesigner.com). Following PCR amplification, free nucleotides were inactivated by shrimp alkaline phosphatase, and amplicons were transcribed *in vitro*, cleaved by RNaseA, and subjected to MALDI-TOF Mass Spectrometry to determine methylation status of the CpGs. EpiTYPER™ software (version 1.0) was used to analyze the results.

### Analysis of *HOXA11* mRNA

The mRNA level of *HOXA11* was analyzed using RT-PCR or quantitative real-time PCR. Total RNA was extracted from cultured cells using RNeasy Mini kit (Qiagen, German), and cDNA was synthesized using the SuperScript™ Ⅲ First-Strand Synthesis System for use with the RT-PCR (Invitrogen) kit, according to the manufacturer's instructions. RT-PCR was carried out in a tube containing 0.5 μg of total RNA and *HOXA11*-specific primers at a final concentration of 0.6 μM using a one step RT-PCR kit (Qiagen, Valencia, CA), according to the manufacturer's protocols. PCR products were quantitated with the GeneAmp PCR System 2700 (Applied Biosystems, Foster City, CA). Quantitative real-time PCR was performed with SYBR Green PCR Master Mix (QIAGEN, Germany) on a LightCyclerⓡ 480 Real-Time PCR System (Roche, Germany). The experiments were performed in triplicate, and results were expressed as mean and standard deviation of the three independent experiments. GAPDH (Glyceraldehyde-3-phosphate dehydrogenase) was used as an internal control to normalize equal amounts of RNA. Primers for RT and real-time PCR were described in Supplementary Table S2.

### Western blot analysis

For Western blot analysis, cells were lysed in PRO-PREP protein extraction solution (iNtRON Biotechnology, Korea), and protein concentrations were determined using the BCA Protein Assay Kit (Pierce, USA). Fifty μg of protein was separated on NuPAGEⓡ 4-12% Bis-Tris Gel (Invitrogen, Carlsbad, CA), and the proteins were electroblotted to polyvinylidene fluoride (PVDF) membranes (Millipore, MA). After blocking with 1% Tween 20-Tris-buffered saline containing 5% nonfat dry milk, the membrane was incubated overnight at 4°C with *HOXA11* antibody (Abcam, England). Membrane was washed three times for 15 minutes with blocking solution and incubated with secondary antibody (Southern biotech, USA) for 2 hours at room temperature. Membrane was washed again three times for 15 minutes with blocking solution and incubated with WEST-ZOL (plus) chemiluminescence reagent (iNtRON Biotechnology, Korea) for 5 minutes and exposed to film (Kodak BioMax light film, USA).

### TSA and 5-Aza-dC treatment of cells

Cells were incubated with 10 μM 5-Aza-2'-deoxycytidine (5-Aza-dC; Sigma Aldrich, St. Louis, MO) for 72 h or were co-incubated with 0.5 μM trichostatin A (TSA) for the final 24 h of those 72 h. After 72 h of culture, cells were harvested and washed in ice-cold PBS. Genomic DNA and mRNA were then isolated and subjected to analysis of the methylation status by EpiTYPER™ assay and methylation-sensitive high-resolution melt (MS-HRM) assay and of the expression level by RT-PCR and quantitative real-time PCR.

### Methylation-sensitive high-resolution melt (MS-HRM) assay

Methylation statuses of *HOXA11* in response to 5-Aza-dC were analyzed using the MS-HRM assay in six lung cancer cell lines, and 10 ng of bisulfite-treated genomic DNA was amplified for the assay. Primers were designed using SEQUENOM EpiDesigner software (Supplementary Table S3), and MS-HRM was performed on LC-480 real-time PCR machine and analyzed by Gene Scanning software (Roche, Swiss).

### Transient transfection of *HOXA11*

To determine the effect of *HOXA11* on migration and proliferation, H23 cells were transfected with 1 μg of *HOXA11* using Lipofectamin™ 2000 (Life Technologies, Carlsbad, CA). Full-length *HOXA11* cDNA (Clone number: IRATp970D0771D) was purchased from Source BioScience (Nottingham, UK). pAcGFP-*HOXA11* constructs were generated by cloning *HOXA11* cDNA in-frame into a pAcGFP1-C1 vector (CLONTECH, Oxford, UK). For the migration assay, H23 cells were seeded in six well plates at a concentration of 1 × 10^5^ cells and incubated overnight to approximately 60-80% confluence. They were then transfected with 1 μg of a pAcGFP1-C1 constitutively expressing *HOXA11* or with an empty vector using Lipofectamin™ 2000 (Life Technologies, Carlsbad, CA) according to the manufacturer's instructions.

After 48 h of transfection, the expression of *HOXA11* was confirmed by western blot analysis according to a standard protocol using primary antibodies directed against GFP (sc-9996; Santa Cruz Biotechnology, CA, USA). Migration assay was performed using transwell migration chambers (6.5 mm diameter polycarbonate membranes, 8 μm pore size) (Corning Costar, Lowell, MA) according to the manufacturer's instructions. The absorbance was measured at 564 nm using VERSAmax microplate reader (Molecular devices). For proliferation assay, H23 cells were seeded into 96-well plates at a density of 1 × 10^3^ after transfection, and cell proliferation was measured by MTT assay every 24 hrs.

### Immunohistochemistry of Ki-67

Ki-67 expression status in formalin-embedded paraffin-embedded tissue samples was assessed as described previously [22]. The fraction of Ki-67-positive cells (Ki-67 proliferation index) was defined as the proportion of the nuclear staining of the tumor cells that were positively stained with a monoclonal anti-Ki-67 antibody (clone MIB-1, DAKO, Carpinteria, CA).

### Methylation-specific polymerase chain reaction (MSP)

The methylation statuses of *HOXA11* in the 317 formalin-fixed paraffin-embedded tissues were determined using methylation-specific PCR (MSP), as described previously (2). For MSP, two sets of primers were used to amplify methylated and unmethylated allele, respectively. The primer sequences are listed in Supplementary Table S4.

### Statistical Analysis

The differences in clinicopathological characteristics and methylation status of *HOXA11* were analyzed using the t-test (or Wilcoxon rank sum test) and the Chi-squared test (or Fisher's exact test) for continuous and categorical variables, respectively. Survival estimate for *HOXA11* hypermethylation on survival was calculated using Kaplan-Meier survival curves, and the statistical comparison of survival curves between the two groups was evaluated using a log-rank test. The hazard ratios of independent predictor variables, after adjusting for potential confounders, were determined using the Cox proportional hazards regression model.

## Supplementary Tables


