# Supplementary material for: HOXA11 hypermethylation is associated with progression of non-small cell lung cancer
Source: Oncotarget. 2013 Oct 28;4(12):2317–25. doi: 10.18632/oncotarget.1464 (PMC3926829; doi:10.18632/oncotarget.1464)
Supplement: Supplementary file 1 [file oncotarget-04-2317-s001.docx]

**Supplementary Table S1. Primer sequences for EpiTYPER^TM^ assay**

| Primer Name | Sequence | Strand | Size | # of CpGs | Tm |
| --- | --- | --- | --- | --- | --- |
| P1-10F | 5’-aggaagagagGAGTTTTGTAAGTAGTTTGATGGGTTT-3’ | + | 263 | 9 | 56.1 |
| P1-T7R | 5-cagtaatacgactcactatagggagaaggctTCATCCCACCTTCTATCCTTAAAA-3’ |  |  |  | 60.1 |
| P2-10F | 5’-aggaagagagGAGGAGGGAGTTTTTTTAAGGAT-3’ | + | 336 | 18 | 59.4 |
| P2-T7R | 5’-cagtaatacgactcactatagggagaaggctCCCCCTCCCATAAACTTACTCTAA-3’ |  |  |  | 60.1 |
| P3-10F | 5’-aggaagagagTTAGAGTAAGTTTATGGGAGGGG-3’ | + | 364 | 23 | 60.1 |
| P3-T7R | 5’-cagtaatacgactcactatagggagaaggctCCACCTCAAAAAAAACAACAAA-3’ |  |  |  | 59.5 |
| P4-10F | 5’-aggaagagagGGAGGTTGGAGAAATTTGGATT-3’ | - | 369 | 12 | 59.4 |
| P4-T7R | 5’-cagtaatacgactcactatagggagaaggctTTCCCCTAAAATAACTACAAAAAAAA-3’ |  |  |  | 58.8 |
| P5-10F | 5’-aggaagagagTTGGTTTTTTTGTTTAAAGGAAGTT-3’ | + | 383 | 16 | 58.7 |
| P5-T7R | 5’-cagtaatacgactcactatagggagaaggctTTATCAACCTAAATCCTAACCACCA-3’ |  |  |  | 60.0 |
| P6-10F | 5’-aggaagagagTTGGATAAAAAGGTTAATTTTGGGT-3’ | - | 441 | 25 | 60.2 |
| P6-T7R | 5’-cagtaatacgactcactatagggagaaggctTATAAAAAACCTTTAAATTTCCCCC-3’ |  |  |  | 59.2 |
| P7-10F | 5’-aggaagagagGTTAGGTGTGGGGGTTGTAATTTAT-3’ | - | 488 | 19 | 60.4 |
| P7-T7R | 5’-cagtaatacgactcactatagggagaaggctACCAAAACTCAAACTAACAAAAACC-3’ |  |  |  | 59.0 |

* F and R indicate forward and reverse primers, respectively.

**Supplementary Table S2. Primer sequences for RT and real-time PCR**

| Genes | Sense | Antisense |
| --- | --- | --- |
| HOXA11 | 5’-CAGCAGAGGAGAAAGAGCGG-3’ | 5’-TGCAGGCGCTTCTCTTTGTTA-3’ |
| GAPDH | 5-‘TGCACCACCAACTGCTTA-3’ | 5’- GGATGCAGGGATGATGTTC-3’ |

**Supplement Table S3. Primer sequences for MS-HRM**

| ID | sequence |
| --- | --- |
| HRM-001-F | TTGAGTATAAGTATGTTGTATGGGGG |
| HRM-001-R | TTCCTTTCTTTATAACCACCTCAAA |
| HRM-002-F | AGTATAAGTATGTTGTATGGGGG |
| HRM-002-R | CCTCAAAAAAAACAACAAATC |

* F and R indicate forward and reverse primers, respectively.

**Supplementary Table S4. Primer sequences for MSP**

|  | Sense | Antisense |
| --- | --- | --- |
| HOXA11_M | 5’- GTTTAGGGTAGGGGGTTTTC -3’ | 5’- CAATCTTTCCCGACTACGAC-3’ |
| HOXA11_U | 5’- GGTTTAGGGTAGGGGGTTTTT-3’ | 5’-CCCAATCTTTCCCAACTACAAC-3’ |

^*^ M and U indicate methylated and unmethylated primers, respectively.
